# Supplementary material for: Preparation, Characterization and Evaluation of Organogel-Based Lipstick Formulations: Application in Cosmetics
Source: Gels. 2021 Jul 19;7(3):97. doi: 10.3390/gels7030097 (PMC8293262; doi:10.3390/gels7030097)
Supplement: Supplementary file 1 [file gels-07-00097-s001.zip › gels-1255378-supplementary.pdf]

Supplementary Material

Preparation, Characterization and Evaluation of Organogel-Based Lipstick Formulations: Application in Cosmetics

Cloé L. Esposito [1] and Plamen Kirilov [2,*]

[1] Faculté de Pharmacie, Université de Montréal, Montréal, QC H3T 1J4, Canada; cloe.esposito@umontreal.ca
[2] Institut des Sciences Pharmaceutiques et Biologiques, Université de Lyon (UCBL), Biologie Tissulaire et Ingénierie Thérapeutique UMR 5305 et Vecteurs Colloïdaux et Transport Tissulaire, 8 Avenue Rockefeller, CEDEX 08, 69373 Lyon, France
* Correspondence: plamen.kirilov@univ-lyon1.fr

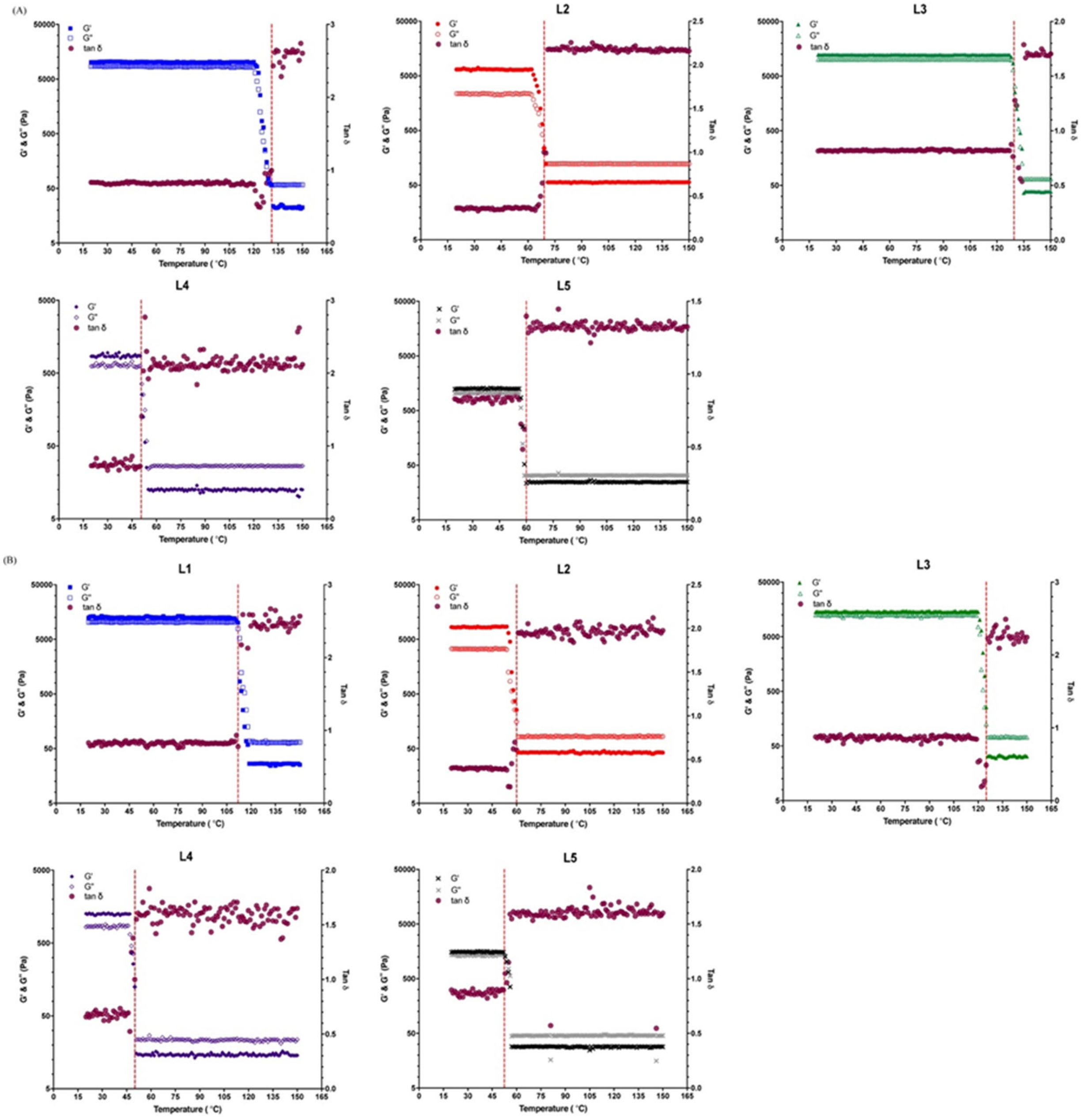

Figure S1. G′, G″ and tan δ versus temperature ramp recorded during the heating of lipsticks, (**A**) of heating sweep and (**B**) of cooling sweep for L1, L2, L3, L4 and L5 formulations. The gel-to-sol transition phase temperature (Tmelt) (corresponding to the point of G′ = G″ and tan δ = 1) are indicated by dashed red lines. Frequencies 1 rad.s^{-1}; 1 C min^{-1} heating rate; a 1% strain within the LVE regime.

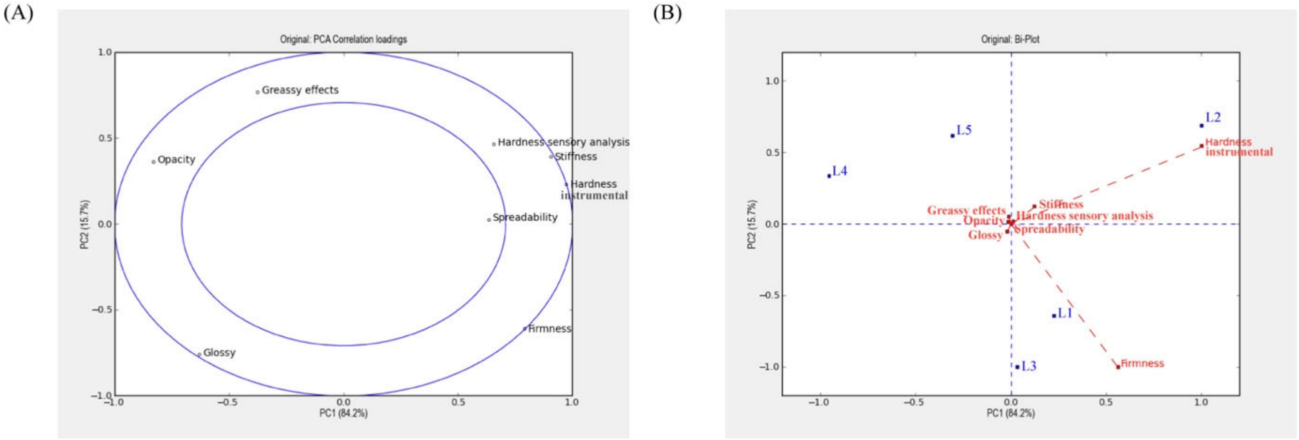

Figure S2. Comparison of sensory attributes and textural instrumental results of the lipstick formulations. Correlation loadings of the principal component analysis (PCA) for all the sensory attributes and texture properties (**A**) and biplot representation of PCA illustrating relationship between sensory attributes, texture measurements and formulations (**B**).

(A)

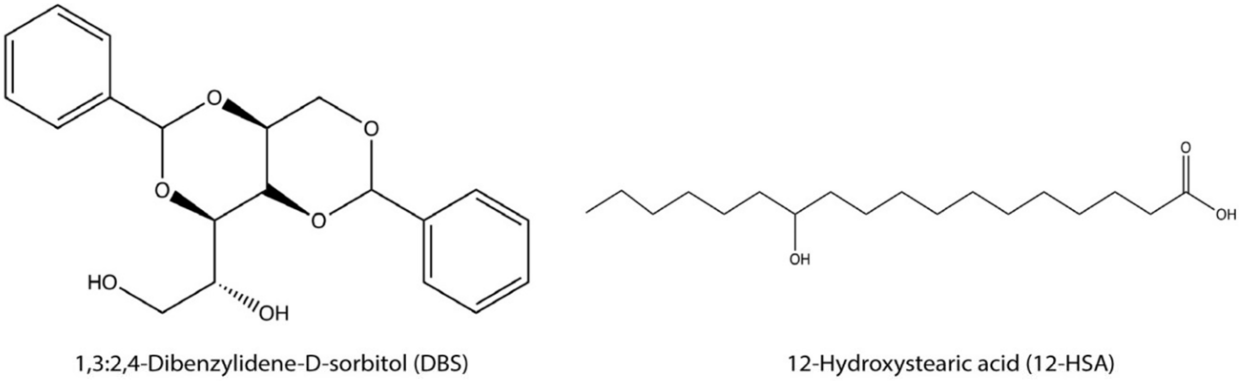

(B)

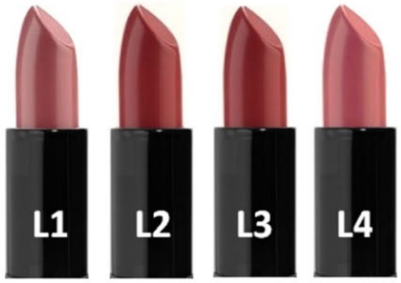

Figure S3. (**A**) Structures of 1,3:2,4-Dibenzylidene-D-sorbitol (DBS) and 12-Hydroxystearic acid (12-HSA) and (**B**) photographs of lipsticks formulations tested in this study.

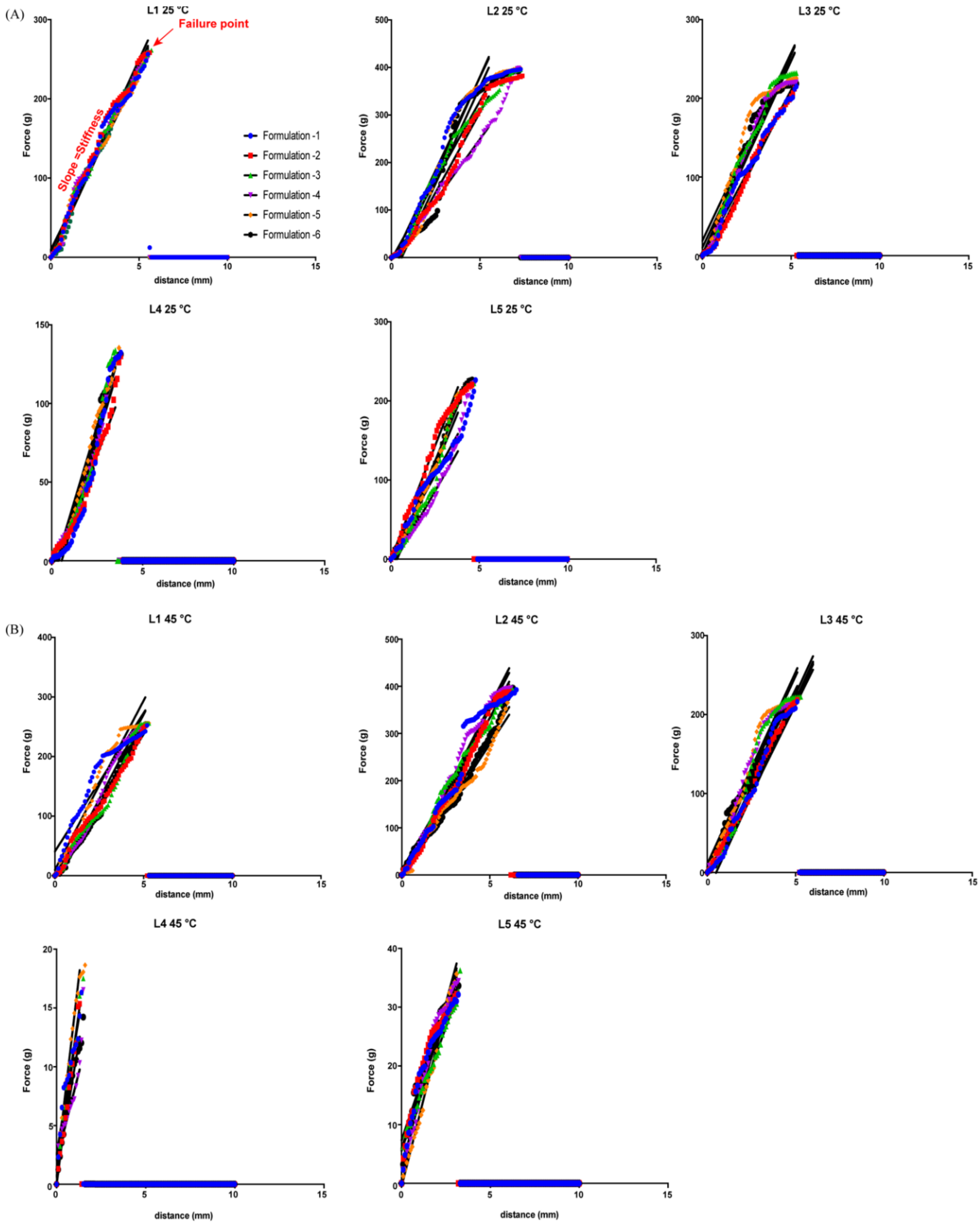

Figure S4. Hardness curves for lipstick formulations at 25 °C (**A**) and 45 °C (**B**) (n = 6 lipsticks per group) showing the gradient of the slope during the bending action referred to "stiffness" of the sample in the elastic region and fracture strength (failure point) at day 1.
